# Defunctioning stoma in anterior resection for rectal cancer does not impact anastomotic leakage: a national population-based cohort study

**DOI:** 10.1186/s12893-023-01998-5

**Published:** 2023-06-20

**Authors:** Eihab Munshi, Marie-Louise Lydrup, Pamela Buchwald

**Affiliations:** 1 Department of Clinical Sciences Malmö, Lund University, Malmö, Sweden; 2 Department of Surgery, University of Jeddah, Jeddah, Saudi Arabia; 3 Department of Surgery, Skåne University Hospital, Malmö, Sweden; 4 Department of Surgery, Samsung Medical Center, Seoul, South Korea

**Keywords:** Rectal cancer, Defunctioning stoma, Defunctioning loop-ileostomy, Anastomotic leakage, Anterior resection

## Abstract

**Background:**

Anterior resection (AR) is considered the gold standard for curative cancer treatment in the middle and upper rectum. The goal of the sphincter-preserving procedure, such as AR, is vulnerable to anastomotic leak (AL) complications. Defunctioning stoma (DS) became the protective measure against AL. Often a defunctioning loop-ileostomy is used, which is associated with substantial morbidity. However, not much is known if the routine use of DS reduces the overall incidence of AL.

**Methods:**

Elective patients subjected to AR in 2007–2009 and 2016-18 were recruited from the Swedish colorectal cancer registry (SCRCR). Patient characteristics, including DS status and occurrence of AL, were analyzed. In addition, independent risk factors for AL were investigated by multivariable regression.

**Results:**

The statistical increase of DS from 71.6% in 2007–2009 to 76.7% in 2016–2018 did not impact the incidence of AL (9.2% and 8.2%), respectively. DLI was constructed in more than 35% of high-located tumors ≥ 11 cm from the anal verge. Multivariable analysis showed that male gender, ASA 3–4, BMI > 30 kg/m^2^, and neoadjuvant therapy were independent risk factors for AL.

**Conclusion:**

Routine DS did not decrease overall AL after AR. A selective decision algorithm for DS construction is needed to protect from AL and mitigate DS morbidities.

**Supplementary Information:**

The online version contains supplementary material available at 10.1186/s12893-023-01998-5.

## Introduction

Anterior resection (AR) is considered the gold standard for the curative treatment of cancer in the middle and upper rectum [1]. The widespread use of total mesorectal excision (TME) and neoadjuvant therapy have improved oncological outcomes and survival [2]. Anastomotic leakage (AL) is a dreaded complication affecting 4–20% of patients undergoing AR [2, 3]. Several risk factors for AL have been described, e.g., male gender, smoking, excess alcohol, overweight, advanced ASA class, diabetes mellitus, pulmonary, renal, vascular diseases, tumor size, neoadjuvant therapy, and anastomotic height from the anal verge [3–5]. Meta-analysis and randomized controlled trials have shown a risk reduction of symptomatic AL after low anterior resection and the need for reoperation in patients with a defunctioning stoma (DS) [6, 7]. Following studies detected a mitigating role of DS in AL complications rather than a decline in overall AL incidence [2, 8]. DS is frequently fashioned as a defunctioning loop-ileostomy (DLI) and more seldom as a loop colostomy [6, 7]. Around 70–80% of AR patients are protected with DS in the UK and Holland, respectively [9, 10]. According to the Swedish colorectal cancer registry (SCRCR), around 600 ARs are performed annually in Sweden [11]. Since most DS are fashioned as DLI, DS will hereafter be named DLI [12, 13].

DLI becomes permanent in about 25% of the cases. The local and systemic physiological changes due to DLI can vary from minor symptoms of skin irritation and leakage (59%) to significant issues like dehydration, obstruction, and parastomal hernia (25%) [14]. One-third of DLI patients risk dehydration in the first six weeks, and half of them require admission for electrolytes correction, possibly putting adjuvant chemotherapy at stake [15]. DLI also impairs health-related quality of life [16]. Delayed DLI closure has been associated with impaired bowel function and major low anterior resection syndrome (LARS) [17, 18]. In addition, about 40% of patients encounter surgical complications, most commonly small bowel obstruction and wound sepsis, during the DLI reversal procedure [14, 19].

This study aimed to evaluate whether the frequency of AL has decreased as the usage of DLI has increased and, as a secondary outcome, investigate risk factors for AL. We hypothesized that the lack of clear indications for DLI leads to increased DLI usage without reducing AL.

## Materials and methods

This study is a population-based retrospective cohort study of patients subjected to rectal cancer surgery in Sweden. The SCRCR is a nationwide registry including rectal cancer patients since 1995 in Sweden with high validity and coverage [20]. We encompassed all rectal cancer patients undergoing AR during two intervals, 2007–2009 and 2016–2018. The criteria for exclusion were age < 18-year-old, emergency surgery, microscopically non-radical resection, and unregistered DLI status. Eligible patients were divided into two groups based on the time for index surgery. We expected patients in the latter group (2016–2018) to be more exposed to DLI than the early cohort (2007–2009) since RECTODES trial results were released in August 2007. Thus, the latter group would be more protected against AL. Each group was further divided into having DLI at the index surgery or not. To be noted, the variable DLI was firstly introduced in the registry in 2007. Patients were discussed at a multidisciplinary team conference.

### Definitions

Rectal cancer was defined as adenocarcinoma within ≤ 15 cm from the anal verge measured with rigid sigmoidoscopy.

According to the International Study Group of Rectal Cancer (ISREC), AL after AR for rectal cancer is defined as a communication between the two cavities (intra- and extraluminal compartments) manifested as a defect in the anastomosis (anastomotic staple or suture line), the presence of a presacral pelvic abscess or a rectovaginal fistula [21] and divided into three grades. AL was reported by different colorectal surgeons in the SCRCR; hence, the validity of the AL definition may vary. In addition, all grades of AL are represented.

DLI is an ileostomy that diverts bowel contents and protects the newly reconstructed colorectal anastomosis applied during the elective AR.

The partial mesorectal excision (PME) variable will be registered in 2022 after the study is completed and the colorectal anastomosis level is not enlisted in SCRCR. Tumor height from the anal verge is employed as a proxy for PME, with PME operations defined as tumors located 11 cm or higher from the anal verge.

### Ethics

Ethical approval was obtained via the Swedish Ethical Review Authority (Diary number 2020 − 01082).

### Statistical analysis

Statistical analyses were made using IBM SPSS version 26. Demographic characteristics were reported as medians with first and third interquartile range (IQR) when continuous and categorical variables as numbers and percentages. Mann-Whitney U test was applied for continuous variables, Fisher’s Exact or Chi-square tests for categorical variables. A two-sided p-value < 0.05 was considered statistically significant. Missing data were reported when exceeding 2% in a variable.

Risk factors for AL were identified using a binary logistic regression model for a univariable and multivariable analysis, including significant univariable variables and other clinically essential indicators like DLI, and index operation date .

## Results

### Study cohort

**Fig. 1:**
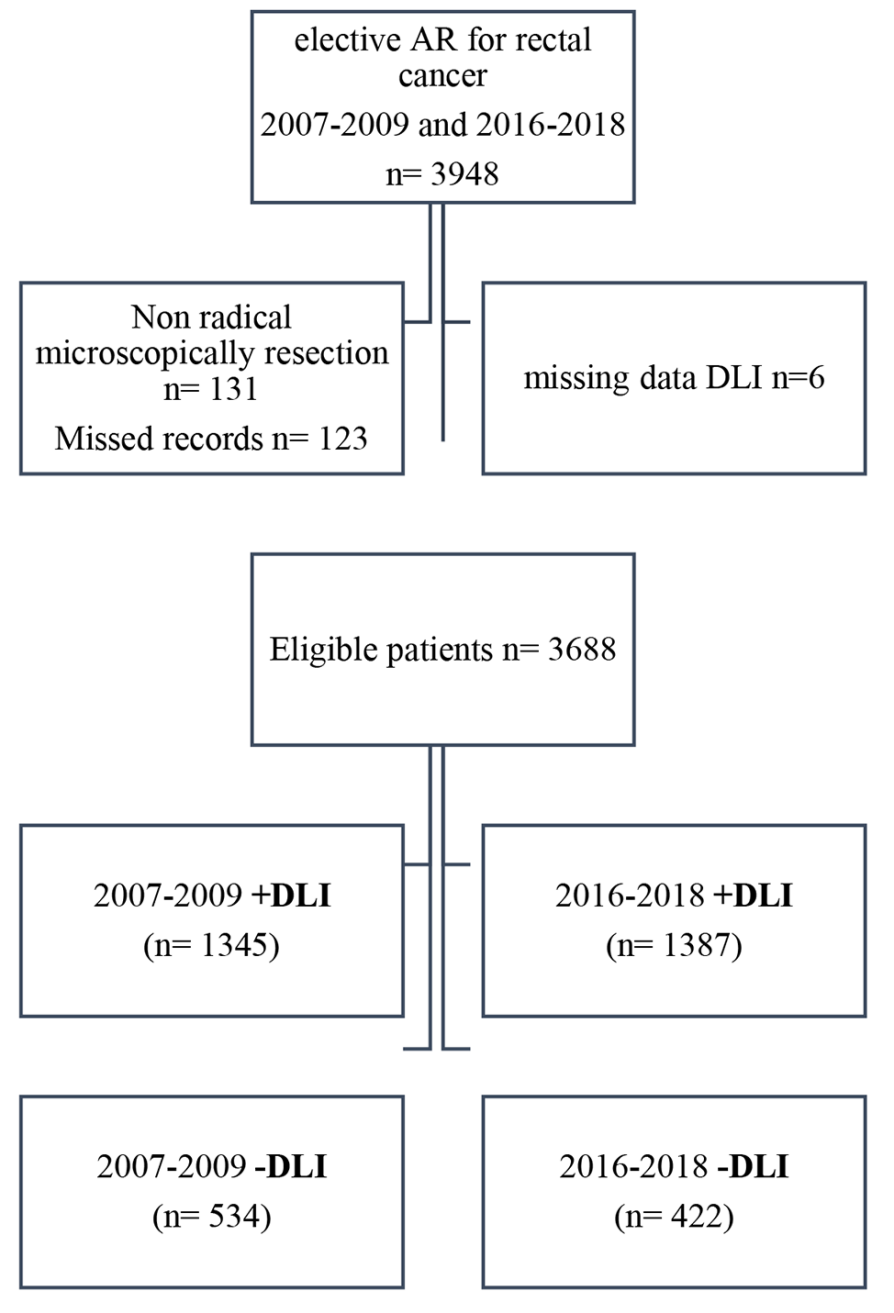
Rectal cancer patients undergoing *AR* (2007-09) and (2016–2018) retrieved from SCRCR and divided into *+/-DLI* *SCRCR*, Swedish Colorectal Cancer Registry, *AR*, anterior resection, *DLI*, defunctioning loop-ileostomy

A total of 3948 AR procedures for rectal cancer were performed during 2007 to 2009 and 2016 to 2018. One hundred thirty-one patients were non-radically resected, 123 patients had missing data regarding resection margins, and six with unregistered DLI status were excluded resulting in 3688 included patients in the study cohort, Fig. 1.

DLI patient characteristics and demographics are displayed in Table 1. Noteworthy, patients in the latter cohort (2016–2018) had more commonly high-located rectal tumor (≥ 11 cm from the anal verge) than the earlier cohort (37% vs. 35%; p < 0.001), and less low-located tumor (< 5 cm from the anal verge) (1.7 vs. 3.4%; p < 0.001). Additionally, less advanced tumor stage and less distant metastasis were found in the latter cohort.

**Table 1:** Comparison of patient characteristics with DLI after AR grouped according to the time (2007–2009) and (2016–2018)

	2007–2009 n = 1345	2016–2018 n = 1387	p-value
**Age (years)**	66 (60–73)	67 (60–73)	0.38^1^
**Gender (male)**	854 (63.5)	852 (61.4)	0.27^2^
**ASA**			**0.001** ^**2**^
ASA1-2	1128 (83.9)	1092 (78.7)	
ASA3-4	189 (14.1)	271 (19.5)	
**BMI** (kg/m^2^)			**0.001** ^2^
<30	1063 (79)	1127 (81.3)	
≥30	165 (12.3)	244 (17.6)	
Missing data	117 (8.7)	16 (1.2)	
Tumor location*			**0.001** ^3^
11–15 cm	474 (35.2)	515 (37.1)	
6–10 cm	799 (59.4)	842 (60.7)	
0–5 cm	64 (3.4)	23 (1.7)	
**pTumor stage**			**0.001** ^**3**^
T0	35 (2.6)	29 (2.1)	
T1-2	479 (35.6)	554 (39.9)	
T3-4	818 (60.8)	803 (57.9)	
Tx	11 (0.8)	0	
**pN stage**			0.17^3^
N0	810 (60.2)	857 (61.8)	
N1-N2	522 (38.8)	523 (37.7)	
Nx	12 (0.9)	5 (0.4)	
**cM stage**			**0.001** ^**3**^
M0	1229 (91.4)	1220 (90.7)	
M1	81 (6)	74 (5.5)	
Mx	33 (2.5)	0	
Missing data	2 (0.1)	93 (6.9)	
**Neoadjuvant therapy**	971 (72.2)	876 (63.2)	**0.001** ^**2**^
**Laparoscopic index AR**	73 (5.4)	815 (58.8)	**0.001** ^**2**^
**High ligation of IMA**	601 (44.7)	766 (55.2)	**0.001** ^**2**^
**Intraoperative perforation**	28 (2.1)	28 (2)	0.953^2^
**Hospital stay (days)**	7 (6–11)	8 (6–13)	**0.001** ^**1**^
**Adjuvant therapy**	398 (29.6)	232 (16.7)	**0.001** ^**2**^
Missing data	22 (1.6)	709 (51.1)	

^1^Mann-Whitney U

^2^Fisher’s exact test

^3^Chi-squared test

*cm for anal verge

*ASA*, American society of anesthesiologists, *BMI*, body mass index, *pT*, pathological tumor stage, *pN*, pathological lymph node stage, *cM*, clinical metastasis, *IMA*, inferior mesenteric artery

Values are shown in numbers and percentages in parentheses for categorical variables. Continuous variables are expressed as median and interquartile ranges

Compared to the former cohort, patients in the latter group comprised more patients with ASA 3–4 (19.5% vs. 14.1%; p < 0.001), BMI > 30 (17.6% vs. 12.3%; p < 0.001), and had a longer hospital stay (8 vs. 7 days; p < 0.001). Throughout the research period, the neoadjuvant therapy was either a short course of 5 × 5 Gy or radio-chemotherapy 2 × 25 Gy with radiosensitizing capecitabine, with the latter group receiving less neoadjuvant therapy (63% vs. 72%; p < 0.001). Moreover, fewer patients in the latter group were treated with adjuvant therapy (17% compared to 39%; p < 0.001). Perioperatively, the latter cohort more frequently underwent minimally invasive anterior resection and high ligation of the inferior mesenteric artery. The characteristics of the PME and TME groups are shown in Supplement Tables 1 and 2.

### Defunctioning loop-ileostomy, anastomotic leakage, and risk factors

Although more than two-thirds of the patients, 71.6% (1345/1879), were diverted by DLI (2007–2009), the diversion rate increased further to 76.7% (1387/1809) (p < 0.001) in the latter group (2016–2018), shown in Table 2.

There was no significant reduction in AL incidence over 11 years despite the expansion in DLI usage (9.2% (124/1879) in 2007–2009 compared to 8.2% (114/1809) in 2016–2018) (p = 0.35). The number of reoperations for AL was unchanged (4% (58/1345) vs. 3% (46/1387), respectively). Comparisons between +/-DLI in the subgroups TME and PME are shown in supplement Tables 1 and 2. The AL rate did not differ between + DLI and -DLI; however, the non-stoma groups needed more reoperations for AL than the + DLI group. This was not significant in the TME group in the latter cohort due to few cases.

**Table 2:** Incidence of AL related to DLI over twelve year-period (2007–2009) and (2016–2018)

	AR 2007–2009 (n = 1879)	AR 2016–2018 (n = 1809)	P-value
DLI	1345 (71.6)	1387 (76.7)	**0.001**
AL	124 (9.2)	114 (8.2)	0.35
Reoperation for AL	58 (4.3)	46 (3.3)	0.17

Fisher’s Exact Test was used

*AR*, anterior resection, *DLI*, defunctioning loop-ileostomy, *AL*, anastomotic leakage

The study cohort was divided into +/-AL, and AL risk factors were analyzed (Table 3). Patients with AL had as many DLI as patients without AL (72% compared to 74%, p = 0.36). In a univariable analysis, AL was significantly related to the male sex, ASA class, BMI, and neoadjuvant therapy.

Male gender, ASA class 3–4, BMI 30 or above, and neoadjuvant therapy remained risk factors for AL in the multivariable analysis (Table 3). DLI and surgery date, on the other hand, had no influence on the incidence of AL.

**Table 3:** Univariable and multivariable analysis for anastomotic leakage risk factors

	Univariable analysis			Multivariable Analysis		
	OR	95% CI	p-value	OR	95% CI	p-value
DLI	0.89	0.69–1.14	0.344	0.84	0.63–1.13	0.252
Operation date (2016–2018)	0.84	0.67–1.05	0.123	0.82	0.65–1.04	0.101
Male sex	1.38	1.09–1.75	**0.008**	1.41	1.09–1.81	**0.008**
ASA 3–4^2^	1.4	1.05–1.82	**0.021**	1.39	1.05–1.85	**0.024**
BMI (> 30 kg/m^2^)	1.5	1.1–1.97	**0.01**	1.51	1.13–2.04	**0.006**
Neoadjuvant therapy	1.27	1–1.6	**0.047**	1.35	1.03–1.75	**0.027**

*ASA*, American society of anesthesiologists, *BMI*, body mass index, *pT*, pathological tumor stage

## Discussion

This study demonstrated a 5% increase in DLI construction between 2007 and 2009 and 2016–2018. Surprisingly, a high number of DLI (71.6%) was registered from 2007 to 2009, although RECTODES-trial was not reported until 2007 [6]. The increased usage of DLI did not reduce AL incidence nor reoperations due to AL. More than one-third of the DLI-patients in both periods had tumors located ≥ 11 cm from the anal verge.

AL is deemed one of the most feared surgical complications after sphincter-preserving surgery. Significant efforts to preclude its occurrence are conducted by minimizing modifiable risk factors and implementing protective measures, including DS. There are inconsistent results on how DS affects AL rates despite well-conducted randomized controlled trials, prospective multicentre studies, and meta-analyses [22, 23]. The present national study indicates that too many AR patients receive a DLI without a beneficial effect on AL, suggesting that the selection process is too blunt. Similarly, a comparison of the Dutch TME-trial in 1996–1999 to the Dutch Surgical Colorectal Audit in 2010 demonstrated significantly increased defunctioning rates from 57 to 70%, albeit AL remained stable (12% vs. 11%) [9].

Additionally, a Swedish regional study found an increase in DS construction from 15% (2002–2006) to 91% (2007–2011), while AL lingered around 10% [24]. The continuing RECTODES trial may have had an impact on clinical practice in both participating and non-participating hospitals. This impact might be attributed to many assumptions, such as DLI structure providing ultimate protection for AL or more frail patients being evaluated for operational care than previously. However, surgeons’ decision-making strategies vary widely, which is a subject for future survey studies. Moreover, the increase in DLI construction would raise the question if some patients would be better off with a permanent colostomy during index surgery and whether some low-risk patients should not be defunctioned, considering comorbidities-related DLI.

The interpretation of the results from RCTs advocating the protective role of DS must consider the circumstances that entail DS construction. In the case of the RECTODEStrial, several detrimental factors were considered. More than two-thirds of AR patients were not accepted for randomization. In our opinion, the most critical exclusion criterion was anastomosis level > 7 cm above the anal verge or resection with a PME procedure. However, in Sweden, a high proportion (25%) of AR patients have high-located tumors (≥ 11 cm from the anal verge), and about 34% subjected to PME were diverted with DLI in Sweden [24, 25]. This high proportion of PME is consistent with our findings which detected a diverting rate of 35% (2007–2009) and 37% (2016–2018).

Furthermore, the most frequent exclusion factor was intraoperative technical difficulties or intraoperative adverse events, which would create a selection bias and, consequently, decrease the external validity.

Blok RD et al. suggested a shift from routine to a highly selective defunctioning ileostomy (HS-DI) after laparoscopic and transanal TME. With a diversion rate of 8% compared to 90% in a historical group, the incidence of AL at 30 days and one year was similar in both groups [26]. Recently, the protocol of the first prospective randomized trial to assess a tailored policy in DS after TME has been presented using an Anastomotic Failure Observed Risk Score (AFOR score) [27].

The short- and long-term stoma-related morbidities for a DS are not negligible. The few weeks after hospital discharge carry a high risk of readmission (17%) due to dehydration by high-output stoma [28]. Moreover, the risk for chronic kidney injury (CKD) accompanying DLI is also time-related, as the incidence of severe CKD injury is higher during the first six months [29]. Stoma reversal is another eventful step that conveys a high rate of 18–40% complications which might require reoperation in 3–8% [30, 31]. Two Swedish population-based cohort studies have investigated the permanent stoma rate, and up to 26% of AR patients would have a permanent stoma. Although AL is one of the most prominent risk factors for the permanent stoma, constructing a defunctioning stoma has no more than negligible effect on maintaining a permanent one [25, 32].

This observational study is strengthened by a large sample representing the national population of this patient group and thus enhances its generalizability and external validity. It was unbiased in selecting all consecutive patients from two periods. However, this registry-based study is limited by unavailable variables. The possibility to explore the effect of DLI on attenuating AL severity and delaying its presentation is hampered by the lack in SCRCR of details on AL presentation and management such as diagnosis date, type of treatment (conservative, antibiotics, drainage, Endoscopic vacuum therapy), details on reoperation, stoma reversal, PME definition and missing data on adjuvant therapy. Another limitation is the unavailability of neoadjuvant treatment toxicity. Neoadjuvant toxicity could be studied for its potential effect on anastomotic healing.

### Conclusion

This population-based study demonstrates an inefficient DLI role in diminishing the risk of AL in routine AR use. Other preventive measures are being studied, including ghost ileostomies, HS-DI, an AL-check list, and scheduled postoperative AL surveillance, including sigmoidoscopy, labs, and rectography. Thereby, an urge for a decision algorithm regarding selective criteria for DLI is called for to spare DLI usage in this complex patient group with multiple risk factors for AL. Noteworthy, there is a significant shift from open to laparoscopic approach in TME-procedure. Therefore, new studies should explore the protective role of DLI specifically in the current surgical practice of laparoscopic and trans-anal TME procedures.

## Electronic supplementary material

Below is the link to the electronic supplementary material.

Supplementary Material 1

## Data Availability

The data that support the findings of this study are available from SCRCR, but restrictions apply to the availability of these data, which were used under license for the current study, and so are not publicly available. Data are however available from the authors upon reasonable request and with permission of SCRCR.
