# Supplementary material for: Defunctioning stoma in anterior resection for rectal cancer does not impact anastomotic leakage: a national population-based cohort study
Source: BMC Surg. 2023 Jun 20;23:167. doi: 10.1186/s12893-023-01998-5 (PMC10283229; doi:10.1186/s12893-023-01998-5)
Supplement: Supplementary file 1 — Supplementary Material 1 [file 12893_2023_1998_MOESM1_ESM.docx]

## **Supplement Table 1.** Patient characteristics in PME and TME patients (with and without DLI) in the first cohort (2007-2009).

|  | TME +DLI  n= 863 | TME  -DLI  n= 125 | p-value | PME +DLI  n= 474 | PME  -DLI  n= 397 | p-value |
| --- | --- | --- | --- | --- | --- | --- |
| **Age (years)** | 66 (59-72) | 73 (64-80) | **<0.001^1^** | 66 (62-73) | 70 (61-77) | **<0.001^1^** |
| **Gender (male)** | 528 (61.2) | 47 (37.6) | **<0.001^2^** | 324 (68.4) | 219 (55.2) | **<0.001^2^** |
| **ASA** |  |  | 0.649^2^ |  |  | 0.222^2^ |
| ASA1-2 | 739 (87.4) | 102 (89.5) |  | 382 (82.3) | 305 (78.8) |  |
| ASA3-4 | 107 (12.6) | 12 (10.5) |  | 82 (17.7) | 82 (21.1) |  |
| **BMI** (kg/m^2^) |  |  | 0.593^2^ |  |  | 1^2^ |
| <30 | 722 (91.5) | 101 (90.2) |  | 382 (88) | 298 (88.2) |  |
| ≥30 | 67 (8.5) | 11 (9.8) |  | 52 (12) | 40 (11.8) |  |
| **pTumor stage** |  |  | 0.44^2^ |  |  | 0.468^2^ |
| T1-2 | 363 (42.5) | 58 (46.4) |  | 149 (31.6) | 134 (34) |  |
| T3-4 | 491 (57.5) | 67 (53.6) |  | 322 (68.4) | 260 (66) |  |
| **pN stage** |  |  | 0.432^2^ |  |  | 0.887^2^ |
| N0 | 509 (59.4) | 78 (63.4) |  | 295 (63) | 247 (63.7) |  |
| N1-N2 | 348 (40.6) | 45 (36.6) |  | 173 (37) | 141 (36.3) |  |
| **cM stage** |  |  | 0.652^2^ |  |  | 0.3^2^ |
| M0 | 797 (95.1) | 112 (94.1) |  | 424 (91.4) | 359 (39.5) |  |
| M1 | 41 (4.9) | 7 (5.9) |  | 40 (8.6) | 25 (6.5) |  |
| **Neoadjuvant therapy** | 694 (80.4) | 64 (51.2) | **<0.001^2^** | 274 (57.9) | 98 (24.7) | **<0.001^2^** |
| **High ligation of IMA** | 386 (44.7) | 37 (29.6) | **<0.001^2^** | 214 (45.1) | 119 (30) | **<0.001^2^** |
| **AL** | 75 (8.7) | 15 (12) | 0.243^2^ | 48 (10.1) | 42 (10.6) | 0.824^2^ |
| **Reoperation for AL** | 37 (4.3) | 14 (11.2) | **0.004^2^** | 21 (4.4) | 35 (8.8) | **0.019^2^** |
| **Accredited colorectal surgeon** | 853 (98.8) | 121 (96.8) | 0.089^2^ | 468 (98.7) | 388 (97.7) | 0.426^2^ |
| **Hospital stay** | 10 (7-15) | 8 (6-13) | **0.006^1^** | 10 (7-15) | 7 (6-10) | **<0.001^1^** |
| **Adjuvant therapy** | 257 (29.8) | 181 (14.4) | **<0.001^2^** | 140 (29.5) | 95 (24) | 0.066^2^ |

^1^Mann-Whitney U

^2^Fisher’s exact test

*ASA*, American society of anesthesiologists, *BMI*, body mass index, *pT*, pathological tumor stage, *pN*, pathological lymph node stage, *cM*, clinical metastasis, *IMA*, inferior mesenteric artery, *AL*, anastomotic leakage.

Values are shown in numbers and percentages in parentheses for categorical variables. Continuous variables are expressed as median and interquartile ranges.

## **Supplement Table 2.** Patient characteristics in PME and TME patients (with and without DLI) in the latter cohort (2016-2018).

|  | TME +DLI  n= 864 | TME  -DLI  n= 101 | p-value | PME +DLI  n= 515 | PME  -DLI  n= 318 | | | p-value |
| --- | --- | --- | --- | --- | --- | --- | --- | --- |
| **Age (years)** | 67 (59-72) | 70 (62-77) | **0.001^1^** | 68 (61-74 ) | 70 (62-76) | | | **0.009^1^** |
| **Gender (male)** | 530 (61.3) | 49 (48.5) | **0.014^2^** | 318 (61.7) | 169 (53.1) | | | **0.017^2^** |
| **ASA** |  |  | **0.023^2^** |  |  | | | 0.568^2^ |
| ASA1-2 | 686 (79.4) | 69 (68.3) |  | 399 (77.5) | 237 (74.5) | | |  |
| ASA3-4 | 164 (19) | 28 (27.7) |  | 107 (20.8) | 76 (23.9) | | |  |
| **BMI** (kg/m^2^) |  |  | 0.492^2^ |  |  | | | 0.565^2^ |
| <30 | 701 (81.9) | 86 (85.1) |  | 422 (82.6) | 262 (84.2) | | |  |
| ≥30 | 155 (18.1) | 15 (14.9) |  | 89 (17.4) | 49 (15.8) | | |  |
| **pTumor stage** |  |  | 0.831^2^ |  |  | | | **0.016^2^** |
| T1-2 | 393 (46.5) | 44 (44.9) |  | 157 (31.2) | 125 (39.7) | | |  |
| T3-4 | 453 (53.3) | 54 (55.1) |  | 346 (68.8) | 190 (60.3) | | |  |
| **pN stage** |  |  | 0.829^2^ |  |  | | | 0.423^2^ |
| N0 | 537 (62.5) | 62 (61.4) |  | 316 (61.6) | 187 (58.8) | | |  |
| N1-N2 | 322 (37.5) | 39 (38.6) |  | 197 (38.4) | 131 (41.2) | | |  |
| **cM stage** |  |  | 0.36^2^ |  |  | | | 0.39^2^ |
| M0 | 770 (95.4) | 94 (96.9) |  | 443 (86) | 290 (91.2) | | |  |
| M1 | 37 (4.6) | 3 (3.1) |  | 36 (7) | 18 (5.7) | | |  |
| Missing | 57 (6.6) | 4 (4) |  | 36 (7) | | 10 (3.1) |  | |
| **Neoadjuvant therapy** | 598 (69.2) | 57 (55.9) | **0.01^2^** | 275 (53.5) | 66 (20.9) | | | **<0.001^2^** |
| **High ligation of IMA** | 481 (55.7) | 53 (52.5) | 0.597^2^ | 283 (55) | 154 (48.4) | | | 0.074^2^ |
| **AL** | 70 (8.1) | 6 (5.9) | 0.56^2^ | 41 (8) | 27 (8.5) | | | 0.796^2^ |
| **Reoperation for AL** | 31 (3.6) | 5 (5) | 0.415^2^ | 15 (2.9) | 24 (7.5) | | | **0.004^2^** |
| **Accredited colorectal surgeon** | 851 (98.5) | 99 (98) | 0.665^2^ | 475 (92.2) | 294 (92.5) | | | 1^2^ |
| **Hospital stay** | 8 (6-14) | 7 (5-11) | **0.004^1^** | 7 (5-13) | 5 (3-8) | | | **<0.001^1^** |
| **Adjuvant therapy** | 142 (16.4) | 19 (18.8) | 0.522^2^ | 89 (17.3) | 25 (7.9) | | | 0.08^2^ |
| **Missing** | 449 (52) | 53 (52.5) |  | 254 (49.3) | 210 (66) | | |  |

^1^Mann-Whitney U

^2^Fisher’s exact test

*ASA*, American society of anesthesiologists, *BMI*, body mass index, *pT*, pathological tumor stage, *pN*, pathological lymph node stage, *cM*, clinical metastasis, *IMA*, inferior mesenteric artery, *AL*, anastomotic leakage.

Values are shown in numbers and percentages in parentheses for categorical variables. Continuous variables are expressed as median and interquartile ranges.
